# Impact of Simulated Reduced-Dose Chest CT on Diagnosing Pulmonary T1 Tumors and Patient Management

**DOI:** 10.3390/diagnostics14151586

**Published:** 2024-07-23

**Authors:** Alan Arthur Peters, Jaro Munz, Jeremias Bendicht Klaus, Ana Macek, Adrian Thomas Huber, Verena Carola Obmann, Njood Alsaihati, Ehsan Samei, Waldo Valenzuela, Andreas Christe, Johannes Thomas Heverhagen, Justin Bennion Solomon, Lukas Ebner

**Affiliations:** 1 Department of Diagnostic, Interventional and Pediatric Radiology, Inselspital, Bern University Hospital, University of Bern, Rosenbühlgasse 27, 3010 Bern, Switzerlandandreas.christe@insel.ch (A.C.);; 2 Carl E. Ravin Advanced Imaging Laboratories, Medical Physics Graduate Program, Clinical Imaging Physics Group, Department of Radiology, Duke University Medical Center, Durham, NC 27705, USA; njood.alsaihati@duke.edu (N.A.);; 3 Department of Diagnostic and Interventional Neuroradiology, Inselspital, Bern University Hospital, University of Bern, 3012 Bern, Switzerland; 4 Department of BioMedical Research, Experimental Radiology, University of Bern, 3012 Bern, Switzerland; 5 Department of Radiology, The Ohio State University, Columbus, OH 43210, USA

**Keywords:** chest, CT scan, lung neoplasms, computer simulation, radiation dosage

## Abstract

To determine the diagnostic performance of simulated reduced-dose chest CT scans regarding pulmonary T1 tumors and assess the potential impact on patient management, a repository of 218 patients with histologically proven pulmonary T1 tumors was used. Virtual reduced-dose images were simulated at 25%- and 5%-dose levels. Tumor size, attenuation, and localization were scored by two experienced chest radiologists. The impact on patient management was assessed by comparing hypothetical LungRADS scores. The study included 210 patients (41% females, mean age 64.5 ± 9.2 years) with 250 eligible T1 tumors. There were differences between the original and the 5%—but not the 25%—dose simulations, and LungRADS scores varied between the dose levels with no clear trend. Sensitivity of Reader 1 was significantly lower using the 5%-dose vs. 25%-dose vs. original dose for size categorization (0.80 vs. 0.85 vs. 0.84; *p* = 0.007) and segmental localization (0.81 vs. 0.86 vs. 0.83; *p* = 0.018). Sensitivities of Reader 2 were unaffected by a dose reduction. A CT dose reduction may affect the correct categorization and localization of pulmonary T1 tumors and potentially affect patient management.

## 1. Introduction

Lung cancer is one of the deadliest cancers worldwide. It accounts for 18% of the cancer deaths overall and is a leading cause of cancer death in men and the second leading cause in women [1].

CT screening examinations enable the detection of small lung cancers in the early stages, improving the patient outcome substantially [2,3]. After detection of the nodules, core-needle biopsy can be used to determine lung cancer types and subtypes, a relatively safe procedure facilitating optimal targeted therapy for each patient [4,5].

Among others, the National Lung Cancer Screening Trial (NLST) found a reduction in mortality rates by 20% in a high-risk population (n = 53,454) when using low-dose CT (LDCT) instead of chest radiography for lung cancer screening (LCS) [6]. Previous investigations also showed that chest radiography is significantly inferior to chest CT examinations regarding the detection of small pulmonary tumors [7,8]. The importance of detecting lung cancers in the tumor stage 1 (T1) is reflected by the 5-year survival rate, which drops from 61% in localized tumor stages to 7% in advanced tumor stages [9]. Therefore, the current study focused on T1 tumors, which by definition are not larger than 3 cm across, have not reached the membranes surrounding the lungs, and do not affect the main branches of the bronchi.

Still, an immanent issue with LCS is the exposure to ionizing radiation. Therefore, the optimization of screening CT protocols plays a key role in the field and has driven numerous investigations in the past. In particular, in the past decade, with the introduction of iterative reconstruction algorithms [10] and highly efficient detector assemblies, LDCT has become a reality that is already in broad clinical use, especially in the field of LCS, with the UK currently leading the way [11,12,13,14].

In the context of LCS, the minimization of radiation exposure is particularly relevant since a large number of healthy individuals without symptoms are exposed. Several studies have addressed this issue in the past using different approaches, and there is a broad consensus that dose reduction is feasible [15,16,17,18].

In this context, LDCT protocols are commonly associated with effective doses of one to two mSv at best [19]. However, recent investigations have shown that further dose reduction is feasible, with an effective radiation dose well below one mSv. Our institution has built a profound expertise regarding ULDCT examinations in phantom studies, which showed that low-dose (1–2 mSv) and ultralow-dose (0.1–0.2 mSv) [20] examinations are feasible for detecting small solid and subsolid nodules [17], with rather high sensitivities and specificities compared to the original dose examinations [21].

Another noteworthy new development in this context is the photon-counting detector CT, as it offers the opportunity to perform chest CT scans at very low doses with minimal noise, retaining a comparable image quality. The first studies regarding lung nodule detection and classification reported very promising results in humans and phantoms [22,23], but further research is warranted, e.g., to rule out potential impacts on patient management recommendations and to elaborate on optimized protocols for indications, such as LCS. However, it underlines the importance of exploring the potential effects of CT dose reduction on pulmonary nodule management.

Several studies have evaluated the effect of dose reduction on the detection of pulmonary nodules in well characterized but relatively small clinical cohorts [24,25], and the validation of those results over a wide variety of different vendors and scanners in a larger clinical cohort is still missing.

This study aimed to evaluate the feasibility of ultra-low dose protocols regarding the detection and classification of histologically proven pulmonary T1 cancers. Unlike previous studies, it utilizes a highly heterogeneous cohort, including data from different sites, including various scan protocols and CT scanner types. Furthermore, it aimed at assessing the potential impact on patient management caused by dose reductions by comparing shifts in the hypothetical Lung CT Screening and Reporting System (LungRADS) scores between the different dose-level groups.

## 2. Materials and Methods

This is a retrospective study with a fully crossed block design with multiple readers and multiple cases. It was approved by the local ethics committee and conducted in accordance with the principles of the Declaration of Helsinki. Only patients with written informed consent were included in the cohort provided by the local lung cancer center (LCC). The authors had full access to and take full responsibility for the integrity of the data.

### 2.1. Study Cohort

The study cohort was based on a repository provided by the local lung cancer center (LCC). It consisted of 218 individuals, all with histologically proven T1 tumors of the lung and available chest CT scans. The examinations were synchronized with the institutional picture archiving and communication system (PACS) and collected in a private user case list. The relevant dose parameters, such as dose-length-product (DLP) or the CT dose index volume (CTDIvol), were documented for each CT scan. Inclusion criteria were as follows: resected pulmonary lesion, histopathologic diagnosis of lung cancer, lesion size <3 cm (T1-stage), preoperative CT-scan present, patient age >18 years. Exclusion criteria were as follows: absence of preoperative CT-scan, higher tumor stages, explicit denial of further data use, insufficient image quality.

The examinations of six patients were deemed ineligible for virtual dose reduction due to excessive image noise (n = 4) and missing CT slices (n = 2). Additionally, two more scans were excluded because of incomplete lung coverage, to avoid potential bias from the smaller scan volume. The characteristics of the included patients (n = 210) and tumors (n = 250) are shown in Table 1.

### 2.2. CT Acquisition and Creation of Virtual (Ultra)Low-Dose Protocols

The 218 CT examinations originated from over 20 different sites all over the country with 5 different CT manufacturers (Siemens, n = 130; Philips, n = 34; GE, n = 28; Toshiba, n = 25; Canon, n = 1). These examinations were conducted over an 8-year period (2010–2018), with the DLP and CTDI values remaining within the respective National Diagnostic Reference Levels [26]. The acquired minimum slice thickness varied from 0.5–3 mm with the vast majority (>80%) being ≤1.5 mm. The reconstruction algorithms contained filtered-back projection (n = 130, 60%), as well as iterative reconstruction (IR, n = 88, 40%). Scan volumes of the included examinations contained whole-body examinations (n = 4, 2%), Positron Emission Tomography and Computed Tomography (PET/CT) scans (n = 36, 17%), chest plus abdomen or neck (n = 46, 22%), and chest-only acquisitions (n = 124, 59%) (Table 2).

Regarding the scan protocol, 62% of the examinations were performed on the local 128-row multidetector Flash CT scanner (Siemens SOMATOM Definition Flash, Siemens Healthineers, Erlangen, Germany) featuring iterative reconstruction algorithms (iterative reconstruction in imaging space (IRIS)) and a detector system with integrated readout electronics, a gantry rotation time of 0.28 s, and a pitch of 2.2. For image reconstruction, an I30f soft tissue kernel was utilized; iterative reconstruction was used to create axial stacks of a 1 mm slice thickness.

The anonymized examinations were transferred to a dedicated post-processing imaging lab specialized in LDCT simulations. The reduced dose simulations were produced by adding statistical noise to the images using a previously described CT image-based noise addition tool [27]. Four reduced dose level simulations were created out of every CT scan, leading to five different dose levels for each examination: 100% (original), 50%, 25%, 10%, and 5% doses.

These simulations were consecutively re-transferred to our institution.

**Table 2:** CT parameters.

Dose parameters of the original scans, mean (SD)	
DLP, mGycm	
Chest only (n = 124)	315.6 (213.5)
Chest plus neck/abdomen (n = 46)	892.6 (557.4)
Whole-body acquisition (n = 4)	540.8 (501.2)
PET-CT (n = 36)	308.6 (155.6)
CTDIvol, mGy	
Chest only (n = 124)	13.7 (13.4)
Chest plus neck/abdomen (n = 46)	27.2 (23.9)
Whole-body acquisition (n = 4)	16.4 (25.1)
PET-CT (n = 36)	4.1 (2.1)
Effective dose, mSv ^#^	
Chest only (n = 124)	4.4 (3.0)
Chest plus neck/abdomen (n = 46)	12.5 (7.8)
Whole-body acquisition (n = 4)	7.6 (7.0)
PET-CT (n = 36)	4.3 (2.2)
Slice thickness, n (%)	
0.5 mm	4 (1.2%)
0.625 mm	3 (0.9%)
0.75 mm	1 (0.3%)
0.9 mm	9 (2.6%)
1 mm	85 (25.0%)
1.25 mm	29 (8.5%)
1.5 mm	12 (3.5%)
2 mm	48 (14.1%)
2.5 mm	2 (0.6%)
3 mm	16 (4.7%)
4 mm	1 (0.3%)

CTDIvol, volume computed tomography dose index; DLP, dose-length-product. ^#^ Calculated from the DLP using the conversion factor of 0.014 [28].

### 2.3. Pilot Study

**Figure 1:**
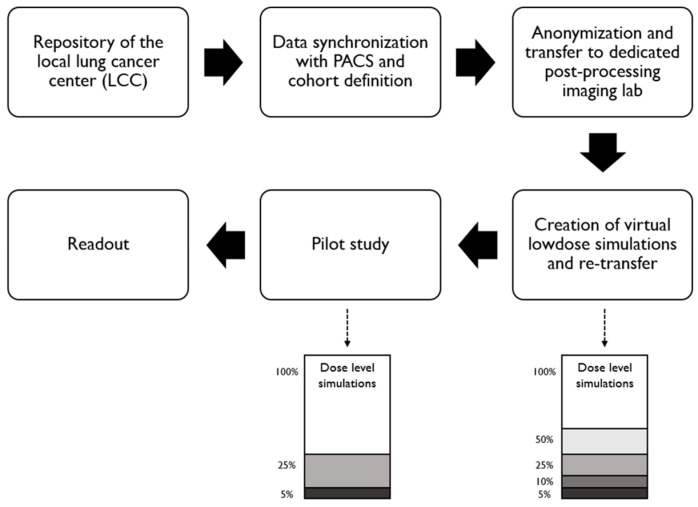
Study workflow.

After preparation of the reduced dose simulations, each reader would have needed to review 1050 examinations (210 × 5), which would have resulted in a highly time-consuming task. Since the differences between adjacent dose levels visually did not seem very striking, a pilot study was conducted in order to compare the signal-to-noise-ratios (SNRs) and contrast-to-noise-ratios (CNRs) of the five dose level groups (study workflow depicted in Figure 1).

Therefore, a board-certified radiologist (AAP) measured the attenuation in Hounsfield units (HUs) and the corresponding standard deviations (SDs) of the air outside the patient (anterior of the sternum), in the bone (central in the vertebral body), and in the soft tissue (autochthonous back musculature) above the level of the diaphragm in 10 randomly chosen patients. SNR was calculated by dividing the signal intensity (SI) of the soft tissue by the background noise (SD of soft tissue SI), CNR by dividing the difference between the SI of bone, and soft tissue by the soft tissue background noise as follows:

$$SNR=\frac{{SI}_{soft\;tissue}}{{SD}_{soft\;tissue}}$$

$$CNR=\frac{{SI}_{bone}-{SI}_{soft\;tissue}}{{SD}_{soft\;tissue}}$$

### 2.4. Readout

Two independent blinded readers (reader 1 with seven years and reader 2 with five years of experience in chest radiology) conducted the readouts of the main study on dedicated workstation monitors (BARCO Coronis Fusion 6MP LED, Kortrijk, Belgium). The readers scored all eligible examinations (3 × 210 = 630 examinations) in a randomized order regardless of the dose level, rating the nodule diameter (based on the categories following LungRADS v2022) and the location and density category (solid, part-solid, ground-glass) of each nodule on a spreadsheet. The readers were allowed to use all kinds of tools, such as multiplanar reformats or maximum intensity projections, in order to read the examinations in the most realistic setting possible. A board-certified radiologist with seven years of experience in chest radiology read all examinations independently and documented the presence of other pulmonary diagnoses, such as emphysema, fibrosis, or pneumonia.

### 2.5. Statistical Analysis

Metric variables are reported as the mean (standard deviation), with categorical variables as absolute numbers (relative percentage). In the pilot study, the SNR and CNR of the different groups were compared using the Friedman test for multiple not normally distributed paired samples with Bonferroni correction for multiple comparisons. For the main study, a generalized linear mixed model (GLMM) with crossed random intercepts for readers and lesions and the dose reduction level as a fixed effect was designed. The binarized endpoints (yes/no) were correct nodule detection, correct categorization of nodule attenuation, size, and localization.

In a subgroup analysis, each reader’s results regarding the respective endpoints for each (simulated) dose level were compared using the Cochran’s Q test.

To assess the possible clinical impact of the findings, the hypothetical LungRADS scores of the tumors were calculated for every dose level. Shifts between the risk groups, which could be assumed to be caused solely by a dose reduction, were documented. The scores were based on LungRADS v2022.

Interrater agreement was assessed by using Cohen’s Kappa (κ). According to Landis and Koch, kappa-values of 0.00 to 0.20 were interpreted as slight, 0.21 to 0.40 as fair, 0.41 to 0.60 as moderate, and 0.61 to 0.80 were interpreted as substantial, while values between 0.81 and 1.00 were interpreted as almost perfect agreement [29].

A *p*-value of <0.05 was considered statistically significant. All analyses were performed using dedicated software: SPSS (SPSS Statistics, IBM Corp., version 25.0. Armonk, NY, USA) and GraphPad Prism (GraphPad Software, Inc., version 8, San Diego, CA, USA).

## 3. Results

**Figure 2:**
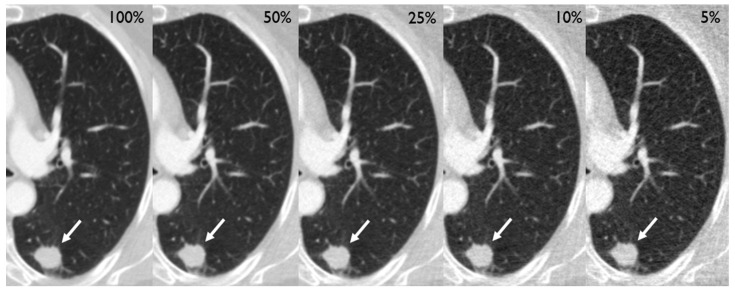
Different dose level simulations depicting a 19 mm primary adenocarcinoma in the left lower lobe of a 71-year old female (white arrow), who had initially presented with a cough.

**Figure 3:**
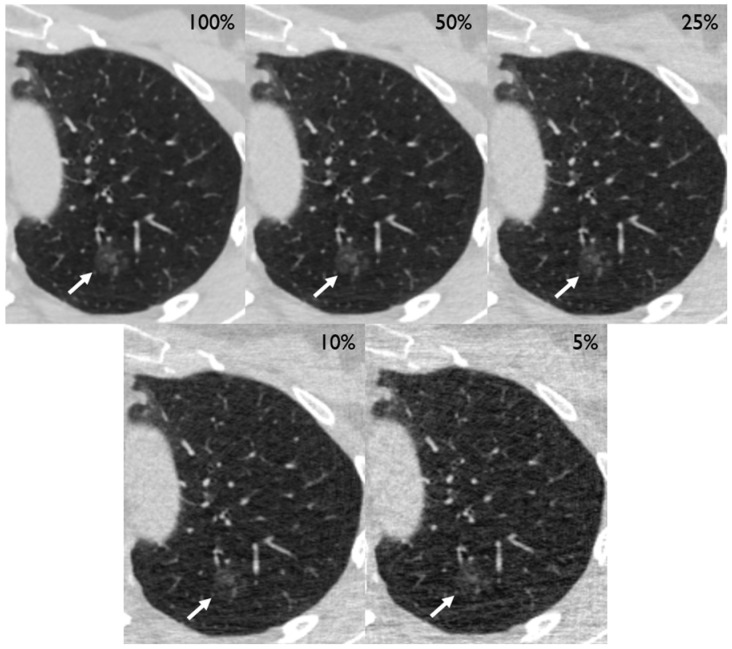
Different dose level simulations depicting a 14 mm ground-glass nodule in the left upper lobe of a 75-year old female (white arrow) with left-sided chest pain. The lesion turned out to be a primary pulmonary adenocarcinoma.

After preparation of the simulations, the CT scans of 210 patients (mean age [SD] 64.5 [9.2] years, 87 females [41%]) containing 250 tumors of the lung (201 solid, 28 part-solid, and 21 ground glass) could be included in the analysis (Table 1). Two exemplary cases are depicted in Figure 2 and Figure 3. Eight patients had to be excluded due to technical reasons, such as incomplete coverage of the lungs.

Regarding contrast media application, 74% (n = 156) of the scans were contrast-enhanced, and 26% (n = 54) were non-contrast scans (Table 2).

### 3.1. Pilot Study

**Figure 4:**
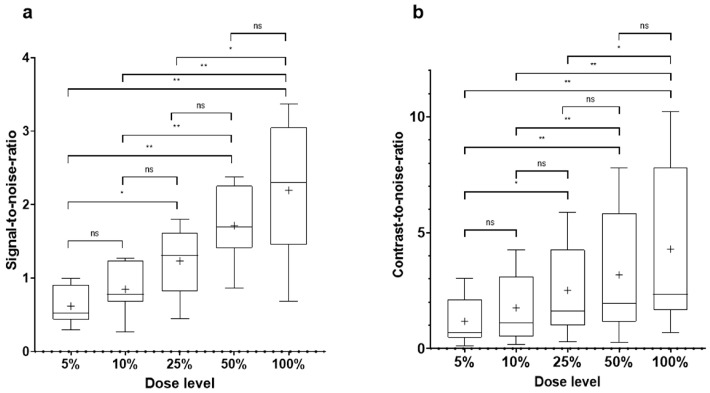
Results of the pilot study: comparison of the different dose level simulations regarding SNR (**a**) and CNR (**b**) depicted as boxplots (min–max). CNR, contrast-to-noise ratio; SNR, signal-to-noise ratio; ns, non-significant; *, significant (*p* < 0.05); **, highly significant (*p* < 0.01).

The comparison of the SNR and CNR of the different dose level simulations in 10 patients revealed that regarding the 10% and 50% simulations, there were no significant differences in the adjacent dose levels (Table 3, Figure 4). Since no significant effects on reader performance were to be expected, the 10% and the 50% dose level simulations were excluded from the further analysis.

### 3.2. Influence of Virtual Dose Reduction on Nodule Detection, Categorization, and Localization

As the main finding, the results of the GLMM indicated no significant differences in the odds of correct nodule detection or correct categorization of nodule size, attenuation, or localization between the different dose levels (Table 4).

#### Subgroup Analysis

In a subgroup analysis by the reader, the comparison of the three dose levels (100%, 25%, and 5% dose simulation) revealed no significant differences regarding the detection rate and false-positive rates (FPRs) for both readers (Table 5a).

**Figure 5:**
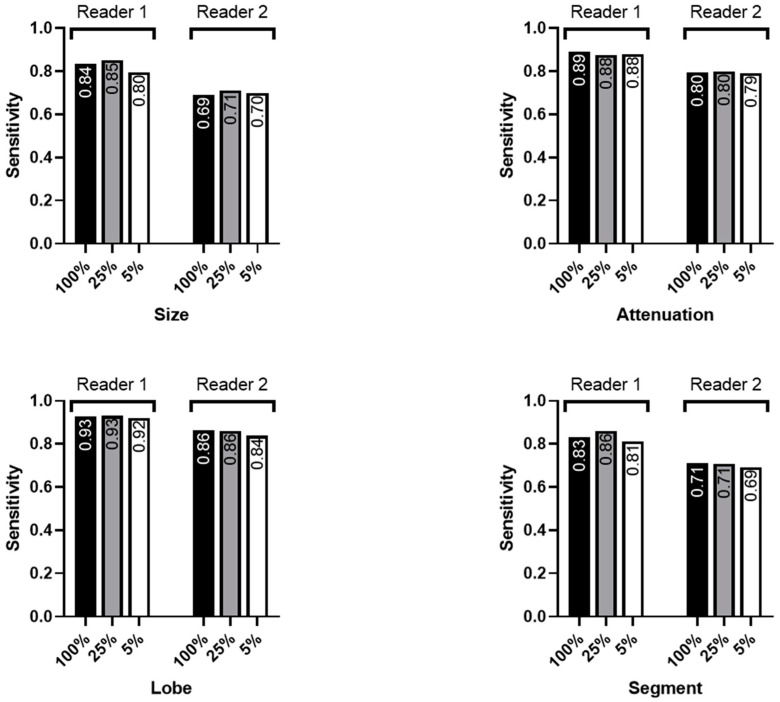
Reader sensitivities regarding the correct categorization of nodule size, nodule attenuation, lobar, and segmental localization by the reader.

Regarding the correct size categorization and segmental localization of the nodules, reader 1 performed significantly inferiorly using the 5%-dose level simulations compared to the higher dose levels. However, reader 2 achieved comparable results for the three dose levels regarding all endpoints (Table 5b, Figure 5).

Regarding the relevant subgroup of subsolid nodules (n = 49), reader 1 achieved sensitivities of 93%, 96%, and 93% for the three dose levels, while reader 2 achieved sensitivities of 86%, 89%, and 86%, respectively.

**Figure 6:**
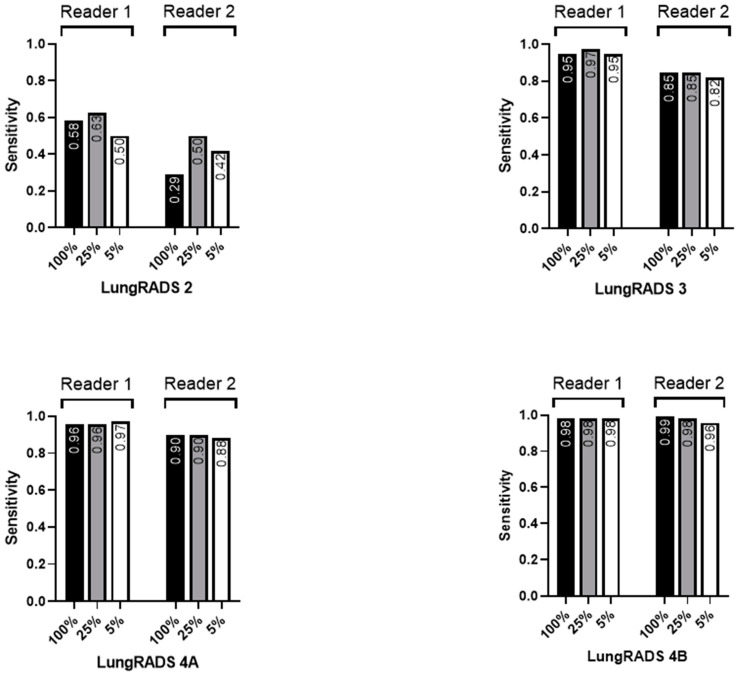
Reader sensitivities regarding nodule detection by LungRADS category.

A subgroup analysis, by LungRADS category, of the tumors was performed and revealed increasing detection rates for both readers with an increasing LungRADS score. Reader 2 detected more LungRADS 2 tumors using the 25%-dose level compared to the original dose level (*p* = 0.022) regarding the smallest subgroup of LungRADS 2 tumors (n = 24). There were no differences between the dose levels for the other LungRADS categories (Figure 6).

Reader 1 was more sensitive regarding the detection of LungRADS 2 tumors (*p* = 0.016), otherwise there were no significant differences between the readers (Figure 6).

There was no significant correlation between detection status and any of the technical or clinical parameters (Table 1).

### 3.3. Impact on Patient Management

Regarding the possible clinical impact of the findings, the hypothetical LungRADS scores of each dose level were compared for each reader. For reader 1, 15% (n = 38) of the tumors shifted LungRADS scores by dose reduction from the original dose to the 25%-dose level, hereby 6% (n = 15) shifted to a lower score, and 9% (n = 23) shifted to a higher score. Dose reduction from the original dose to the 5%-dose level led to a shift for 17% of the tumors (n = 42), 6% (n = 15) to a lower score, and 11% (n = 27) shifted to a higher score. 

The corresponding value for reader 2 regarding the dose reduction from the original dose to the 25%-dose level was 18.0% (n = 45), of which 9% (n = 22) shifted to a lower and 9% (n = 23) to a higher risk score. After reducing the original dose to the 5%-dose level, 20% (n = 50) of the tumors shifted overall, hereby 10% (n = 24) shifted to a lower and 10% (n = 26) to a higher risk score.

### 3.4. Interrater Agreement

Regarding the hypothetical LungRADS categorization of the nodules (comprising nodule size and density), the readers showed a moderate agreement for all dose levels (κ = 0.459–0.492). Following the definition of Landis and Koch [29], the analysis revealed almost perfect interrater agreement regarding lobar localization (κ = 0.824–0.857) and substantial agreement regarding segmental localization (κ = 0.661–0.700) of the nodules for all dose levels (Table 6).

## 4. Discussion

This study analyzed the impact of dose reduction on the detection, localization, categorization, and management of pulmonary T1 tumors by using virtual (ultra)low-dose CT protocols.

The main finding of this study was that dose reduction in chest CT is feasible regarding pulmonary nodule detection, localization, and classification. However, according to the subgroup analyses, tumor localization and size categorization might be affected by a dose reduction for certain readers. Fletcher et al. analyzed the detectability of pulmonary nodules in the chest CT scans of 21 patients containing 28 nodules using five different dose levels and found that dose reductions by 70% or more are non-inferior compared to the routine clinical dose levels [16]. In a follow-up study, they revealed that scanning pulmonary nodules 5 mm or larger at very-low-dose levels are feasible down to a 10-quality reference mAs (QRM) level but might lead to a loss of detection regarding a significant proportion of part-solid nodules [24].

In the current study, there were no differences regarding the detection rates of part-solid nodules between the different dose levels; however, it has to be mentioned that the number of included part-solid nodules was rather small (n = 28).

The mean CTDIvol in this study was relatively high compared to similar studies, most probably because the included examinations comprised not only chest CT scans but also abdominal or whole-body scans acquired in various clinical settings [24]. However, the effectively applied doses were in similar ranges [30].

Nodule detection rates of both readers were excellent for all dose levels while maintaining an acceptable FPR (range: 0.13–0.45).

In order to evaluate the secondary endpoint, readers had to localize the nodules correctly and categorize them by size and attenuation; the categories were defined in accordance to LungRADS v2022 in order to assess the potential impact on patient management. LungRADS categories shifted between the different doses, indicating a potential impact on patient management. After calculation of the hypothetical LungRADS scores, 15.2% and 16.8% of all tumors would have shifted to a different LungRADS category after dose reduction from the original dose to the 25%- and 5%-dose level, respectively, for reader 1. The corresponding values for reader 2 were even a bit higher with 18.0% and 20.0%. Taking into regard a measurement variability of 25% reported by several in vivo “coffee-break” studies and the proportion of differing LungRADS scores between two different computer-aided diagnosis (CAD) systems measuring the same nodule of approximately 15%, these values seem acceptable and are in line with the literature [31,32,33].

In a similar study, Paks et al. compared an LDCT protocol to an ULDCT protocol regarding pulmonary nodule detection and volumetry in 188 solid pulmonary nodules greater than 2 mm and concluded that ULDCT delivers comparable results and therefore may be used for follow-up examinations [30]. However, they did not assess the impact on patient management specifically, limiting the comparison to the current study.

Hata et al. and Milanese et al. both assessed the impact of dose reduction on LungRADS classification by radiologists [34,35]. While Milanese et al. found excellent intraobserver agreement between low dose and ultra-low dose scans, Hata et al. reported varying agreements between the original dose and reduced dose scans, indicating a potential impact of dose reduction on the LungRADS classification, which is in line with the current results. However, it should be noted that in the latter study, the median volume of the nodules was approximately 50 mm³, compared to volumes ranging from 75 mm³ to 194 mm³ in Milanese et al., which made interpretation more challenging. 

A task for future studies will be the evaluation of (AI-) CAD systems in this context, since such tools are broadly utilized in daily clinical routines in order to support radiologists, who are confronted with an increasing workload and benefit from CAD systems, especially if used as second reader devices [36,37,38]. Regarding the current study, it can be hypothesized that the use of (AI-) CAD systems potentially could have enhanced the readers’ performance, especially reader 1 might have had benefitted from a second reader device. In theory, the use of CAD systems leads to more robust and reproducible readout results. 

Interestingly, it could be shown that CT dose reduction has an influence on the performance of a deep learning (DL)-based CAD system regarding malignancy prediction in a high-risk cohort of proven malignancies [39]. This finding implies that the CAD systems still require radiological supervision as their performance is dependent on image quality.

For daily clinical routines, these results imply increased awareness while reading reduced-dose chest CT scans, for instance in the context of LCS. This applies to both readouts performed by radiologists alone and AI-assisted readouts. If CAD systems are utilized, the results demand supervision by a radiologist in a second reader scenario, since the risk of lung cancer under- or overestimation and all the associated, potentially unnecessary consequences, such as biopsies, is given.

This study has limitations. First, although the results of the current study indicated no statistical difference between the different dose level scans, the design did not allow for any statement on the non-inferiority of the reduced-dose CT scans, which should be targeted in follow-up studies.

Second, the virtual reduced-dose protocols were created during post-processing and are not perfectly transferable to true ULDCT protocols. However, this approach was the most realistic and at the same time ethically justifiable one. Third, CT scans from various institutions and vendors were included with differing settings, which may have affected the quality of the simulations. On the other hand, this approach led to robust and generalizable results. Fourth, there were only a small number of part-solid nodules included in this study. This type of nodule is very relevant, since it has a higher probability of malignancy and is specifically vulnerable to higher image noise. However, the management defining and therefore most relevant part of the nodule is the solid portion, which was evaluated by the readers. Fifth, the results were based on the readout results of only two readers resulting in limited generalizability and demanding follow-up studies with a higher number of readers. Lastly, there was no washout period for the readers between the readout of the different dose levels to definitely rule out recall bias. However, randomization of the large number of cases prevented an order effect.

In conclusion, the results of this study indicate that a dose reduction to 25% or 5% of the original dose is feasible for the detection and localization of pulmonary T1 cancers. Alterations to patient management based solely on dose reduction cannot be ruled out by the current results; however, there is no clear tendency towards malignancy risk over- or underestimation. For clinical routines, respective measures should be taken to address this problem, for instance the utilization of a second reader setup.

## Figures and Tables

**Table 1:** Patient and nodule characteristics.

Sex (f/m)	87/123
Age [Years, Mean (SD)]	64.5 (9.2)
Other pulmonary diagnoses, n (%)	
Emphysema	120 (57.1%)
Fibrosis	2 (1.0%)
Pulmonary congestion	8 (3.8%)
Pleural effusion	9 (4.3%)
Pneumonia	28 (13.3%)
Atelectasis	21 (10.0%)
Bronchitis	157 (74.8%)
SAD	28 (13.3%)
Postoperative status	5 (2.4%)
Nodule localization, n (%)	
Upper lobe	133 (53.2%)
Middle lobe/lingula	17 (6.8%)
Lower lobe	100 (40.0%)
Nodule attenuation and diameter, n (%)	
Solid	201 (80.4%)
<4 mm	3
4–6 mm	11
>6–8 mm	19
>8–15 mm	50
>15–30 mm	118
Part-solid	28 (11.2%)
<6 mm	1
≥6 mm	27
Ground glass	21 (8.4%)
<30 mm	21
>30 mm	0
LungRADS category, n (%)	
2	24 (9.6%)
3	39 (15.6%)
4A	69 (27.6%)
4B	118 (47.2%)

SAD, small airway disease.

**Table 3:** SNR and CNR by dose level (n = 10).

Dose level	100%	50%	25%	10%	5%
Noise, mean (SD)	22.4 (6.8)	29.3 (10.1)	39.9 (13.3)	61.1 (25.1)	86.0 (34.5)
SNR, mean (SD)	2.2 (0.9)	1.7 (0.5)	1.2 (0.4)	0.8 (0.3)	0.6 (0.3)
CNR, mean (SD)	4.3 (3.6)	3.2 (2.7)	2.5 (2.0)	1.8 (1.5)	1.2 (1.0)

CNR, contrast-to-noise ratio; SNR, signal-to-noise ratio.

**Table 4:** Results of the generalized linear mixed model.

Variable	Terms	Odds Ratio	95%-CI	*p*-Value
Detection	Original vs. 25%	1.23	0.73–2.28	*0.385*
	Original vs. 5%	0.86	0.50–1.48	*0.579*
Size	Original vs. 25%	1.16	0.81–1.67	*0.408*
	Original vs. 5%	0.88	0.62–1.25	*0.472*
Attenuation	Original vs. 25%	0.93	0.61–1.42	*0.747*
	Original vs. 5%	0.91	0.60–1.39	*0.668*
Localization	Original vs. 25%	1.12	0.77–1.64	*0.559*
	Original vs. 5%	0.83	0.57–1.21	*0.341*

CI, confidence interval.

**Table 5:** (**a**). Nodule-based detection sensitivity and FPR by dose level. (**b**). Correct nodule categorization and localization by dose level (n = 250).

(a)					
	Dose Level	100%	25%	5%	*p*-Value ^a^
Reader 1	Sensitivity, n (%)	236 (94%)	238 (95%)	235 (94%)	*0.097*
	Sensitivity (part-solid), n (%)	26 (93%)	27 (96%)	26 (93%)	*0.368*
	FPR, n ($\frac{n}{210}$)	94 (0.45)	87 (0.41)	86 (0.41)	*0.543*
Reader 2	Sensitivity, n (%)	223 (89%)	229 (92%)	218 (87%)	*0.419*
	Sensitivity (part-solid), n (%)	24 (86%)	25 (89%)	24 (86%)	*0.717*
	FPR, n ($\frac{n}{210}$)	27 (0.13)	28 (0.13)	32 (0.15)	*0.607*
**(b)**					
	**Dose Level**	**100%**	**25%**	**5%**	***p*** **-Value ^b^**
Reader 1	Size	0.84	0.85	0.80	*0.007* *
	Attenuation	0.89	0.88	0.88	*0.161*
	Lobe	0.93	0.93	0.92	*0.325*
	Segment	0.83	0.86	0.81	*0.018 ^#^*
Reader 2	Size	0.69	0.71	0.70	*0.798*
	Attenuation	0.80	0.80	0.79	*0.942*
	Lobe	0.86	0.86	0.84	*0.442*
	Segment	0.71	0.71	0.69	*0.761*

FPR, false-positive rate. ^a^ Cochran’s Q test or Friedman test, as appropriate. ^b^ Cochran’s Q test. * Post-hoc-analysis: 100% vs. 25%, *p* = 0.401; 100% vs. 5%, *p* = 0.036; 25% vs. 5%, *p* = 0.003. ^#^ Post-hoc-analysis: 100% vs. 25%, *p* = 0.105; 100% vs. 5%, *p* = 0.247; 25% vs. 5%, *p* = 0.005.

**Table 6:** Interrater agreement.

	Dose Level	100%	25%	5%
LungRADS score * (κ)		0.459	0.492	0.465
Lobe (κ)		0.857	0.839	0.824
Segment (κ)		0.700	0.688	0.661

* LungRADS score comprises nodule size and attenuation (based on LungRADS v2022).

## Data Availability

Research data is available upon specific request from the authors.

## Abbreviations

CNR	Contrast-to-Noise Ratio
CTDI	Computed Tomography Dose Index
DLP	Dose Length Product
FPR	False Positive Rate
HU	Hounsfield Unit
IR	Iterative Reconstruction
LCC	Lung Cancer Center
LCS	Lung Cancer Screening
LungRADS	Lung CT Screening and Reporting System
NLST	National Lung Cancer Screening Trial
PACS	Picture Archiving and Communication System
PET/CT	Positron Emission Tomography and Computed Tomography
SD	Standard Deviation
SNR	Signal-to-Noise Ratio
Sv	Sievert
T1 (a, b)	Tumor stage 1 (a, b)
(U)LDCT	(Ultra)low-dose Computed Tomography
